# Equilibria between conformational states of the Ras oncogene protein revealed by high pressure crystallography†

**DOI:** 10.1039/d1sc05488k

**Published:** 2022-01-13

**Authors:** Eric Girard, Pedro Lopes, Michael Spoerner, Anne-Claire Dhaussy, Thierry Prangé, Hans Robert Kalbitzer, Nathalie Colloc'h

**Affiliations:** Univ. Grenoble Alpes, CEA, CNRS, IBS Grenoble France; Institute of Biophysics and Physical Biochemistry, Centre of Magnetic Resonance in Chemistry and Biomedicine, University of Regensburg Regensburg Germany hans-robert.kalbitzer@biologie.uni-regensburg.de; Normandie Univ., Ensicaen, CNRS, CRISMAT UMR 6508 Caen France; CiTCoM UMR 8038, CNRS Université de Paris, Faculté de Pharmacie Paris France; ISTCT UMR 6030, CNRS, Université de Caen Normandie, CERVOxy Group, Centre Cyceron Caen France colloch@cyceron.fr

## Abstract

In this work, we experimentally investigate the allosteric transitions between conformational states on the Ras oncogene protein using high pressure crystallography. Ras protein is a small GTPase involved in central regulatory processes occurring in multiple conformational states. Ras acts as a molecular switch between active GTP-bound, and inactive GDP-bound states, controlling essential signal transduction pathways. An allosteric network of interactions between the effector binding regions and the membrane interacting regions is involved in Ras cycling. The conformational states which coexist simultaneously in solution possess higher Gibbs free energy than the ground state. Equilibria between these states can be shifted by applying pressure favouring conformations with lower partial molar volume, and has been previously analyzed by high-pressure NMR spectroscopy. High-pressure macromolecular crystallography (HPMX) is a powerful tool perfectly complementary to high-pressure NMR, allowing characterization at the molecular level with a high resolution the different allosteric states involved in the Ras cycling. We observe a transition above 300 MPa in the crystal leading to more stable conformers. Thus, we compare the crystallographic structures of Ras(wt)·Mg^2+^·GppNHp and Ras(D33K)·Mg^2+^·GppNHp at various high hydrostatic pressures. This gives insight into per-residue descriptions of the structural plasticity involved in allosteric equilibria between conformers. We have mapped out at atomic resolution the different segments of Ras protein which remain in the ground-state conformation or undergo structural changes, adopting excited-energy conformations corresponding to transient intermediate states. Such *in crystallo* phase transitions induced by pressure open the possibility to finely explore the structural determinants related to switching between Ras allosteric sub-states without any mutations nor exogenous partners.

## Abstract 2

The equilibria between structural states induced by pressure within the crystal structure of Ras are illustrated with different colors corresponding to different Ras substates.

## Graphical abstract

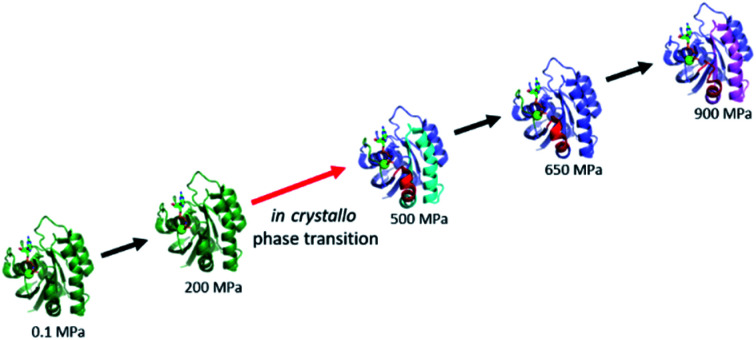

## Introduction

Proteins in solution equilibrate among multiple conformational states, constituting what is termed their conformational energy landscape. These various states encompass all functional substates that are biologically relevant as well as folding intermediates.^1,2^ They are called high-energy conformers or excited states, with Gibbs free energy being higher than the native ground state. These substates are required to allow the protein to interact with its different partners. Under normal conditions, high-energy conformers are weakly populated and therefore difficult to detect.

To explore the conformational space available to proteins and to catch excited states, it is necessary to modify their thermodynamic environment. To achieve that, pressure is a powerful tool. Indeed, thermodynamic conjugate of pressure is volume, which is directly related to the three-dimensional structure and to conformational changes of a protein necessary for its proper function and interactions with other proteins or small ligands. By applying higher and higher pressures, one can increasingly populate conformers with higher Gibbs free energies and smaller partial molar volumes. Pressure allows to identify specific conformational states and to characterize shifts in the population of these states. Pressure can even lead to an inversion of the distribution between the basic folded and excited states.^3–7^ The effect of high hydrostatic pressure was investigated either by NMR^8–13^ (HP-NMR) or by macromolecular crystallography^14–18^ (HPMX), to explore the conformational space and unfolding pathways, to characterize shifts in the population of sub-states, and to map flexibility and rigidity relevant for functional efficiency.

Ras protein is a small GTPase involved in central regulatory processes such as cell differentiation, proliferation and apoptosis. It possesses multiple conformational states and acts as a molecular switch between an active, GTP-bound state, and an inactive, GDP-bound state, controlling essential signal transduction pathways. Ras displays a slow intrinsic activity for GTP hydrolysis that is accelerated by GTPase activating proteins (GAP) by several orders of magnitude. Similarly, the exchange of GDP to GTP requires guanine nucleotide exchange factors (GEF) for accelerating this process. Active Ras interacts also with effectors, through which the different signalling cascades are activated.^19–21^ Specific somatic mutations render Ras a very potent oncogene. Particularly, substitutions at the amino acid positions Gly 12, Gly 13 and Gln 61 lead to a constitutive activation leading ultimately to uncontrolled cellular proliferation. In fact, approximately 30% of all tumours are estimated to be Ras-driven.^22^ Ras is anchored to the membrane *via* its C-terminal domain, and activated through cell surface receptors.^23–28^ Ras is part of a very complex network which requires the coexistence of multiple conformational states through which the protein needs to interconvert very rapidly. These states are termed (T) or (D), depending on whether nucleoside tri or diphosphates are bound. Two segments of Ras termed switch I and switch II correspond to the regions that exhibit large conformational changes upon transition from the GTP- to the GDP-bound state. These two switch regions interact with the Ras-binding domains (RBDs) of downstream effector proteins such as Raf kinase, PI3K and RalGDS, leading to activation of the respective signalling pathways, and also with GAPs and GEFs, that switch off or on the active state of Ras conformations.

Protein–protein interactions in the Ras pathway are transient and relatively weak, and thus difficult to characterize. The dynamics of H-Ras (amino acids 1 to 166) have been investigated by liquid-state ^31^P NMR spectroscopy and by solid-state ^31^P MAS NMR of a powder of single crystals of Ras·Mg^2+^·GppNHp.^29–32^ By using the bound nucleotide as probe, two conformational states, named state 1(T) and state 2(T), that exchange in the millisecond scale could be directly observed. State 1(T), weakly populated, corresponds to the GEF recognition state while state 2(T), the dominant state, represents the high-affinity binding state for effectors.

HP-NMR spectroscopy using ^15^N enriched Ras(wt) allowed to detect and thermodynamically to characterize four functional states. In addition to states 1(T) and 2(T), a GAP-interacting state 3(T) and a nucleotide releasing state 1(0) were identified.^10,12^ The population of state 2(T) decreases with pressure while the population of the other states initially increases. State 1(T) has a maximum population at 40 MPa. At approximately 80 MPa the populations of states 1(T), 2(T) and 3(T) are almost equal, at 180 MPa, state 3(T) reaches its maximum and becomes the prominent state. At pressures higher than 400 MPa, Ras is predicted to exist almost exclusively in the nucleotide releasing state 1(0) with low affinity for the Mg^2+^-complexes of the nucleotides. The conformations identified by HP-NMR are supposed to represent the structures relevant for a conformational selection mechanism in protein–protein recognition and to be somewhat modified by an induced fit after the complexes are formed.

Ras structure consists of a central β-sheet core (β1–β6) surrounded by a bundle of 5 α-helices (α1–α5). In the state 2(T), an allosteric network has been described between the effector lobe, containing the active site and the effector binding regions, and the C-terminal allosteric lobe, containing the membrane interacting regions, through many direct and water-mediated connections.^25,26,33–35^ This allosteric network regulates the interaction of Ras with effectors, with a transition between two states 2(T), termed “on” and “off”. In the “on” state, the allosteric switch (composed of the α3 helix and the loop β5) is shifted toward the α4 helix and the switch II is ordered with the residue Gln 61 ready for catalysis, while in the “off” state, the allosteric switch is shifted toward the switch II, inducing the disorganization of its N-terminal portion. Another state has been described for the mutant Q61L, the “ordered off” state, where the allosteric switch is in the “off” state while the switch II remains ordered.^36,37^

The crystal structures of Ras(wt) correspond mainly to state 2(T), which is the dominant state at ambient pressure.^38–40^ Crystal structures of different Ras mutants like Ras(T35S) and Ras(G60A), or glue-coated Ras(wt) prepared in humid air (HAG) have been proposed as representatives of state 1(T).^41–43^ Differences between states 1(T) and 2(T) reside mainly in the switch I region, with the Thr 35 hydroxyl group coordinated to the Mg^2+^-ion in state 2(T) but not in state 1(T).

As observed also for the Ras-binding domain of Raf^44^ the binding of Ras(wt) in state 2(T) to the regulatory Ras-binding domain (RBD) of GEF is accompanied by an induced-fit process dependent on the surface of the RBD. A ternary complex of the nucleotide exchange factor SOS with Ras(Y64A)·Mg^2+^·GppNHP in a regulatory site and on the catalytic side a nucleotide-free Ras could be a possible representative of a state 2(T)*-RBD and a nucleotide-free state 1(0) respectively.^45^

Contrary to the Ras(wt)·Mg^2+^·GppNHp behaviour, the mutant Ras(D33K)·Mg^2+^·GppNHp shows a very low population of state 1(T) in solution, according to its ^31^P NMR spectrum. Its crystal structure was unknown prior to this work. Ras·Mg^2+^·GppNHp and Ras(D33K)·Mg^2+^·GppNHp will be referred in the following as Ras(wt) and Ras(D33K).

To precisely characterize the pressure-induced modification of equilibrium previously described, we have pressurized crystals of Ras(wt) and of Ras(D33K) up to 900 MPa. We have thus detected equilibrium between conformers within the crystalline state and identify step-by-step the protein segments that drive the transition between states. This approach allows us to probe the conformational allosteric landscape of Ras.

## Results

### Conformational equilibria in Ras(D33K)

**Fig. 1:**
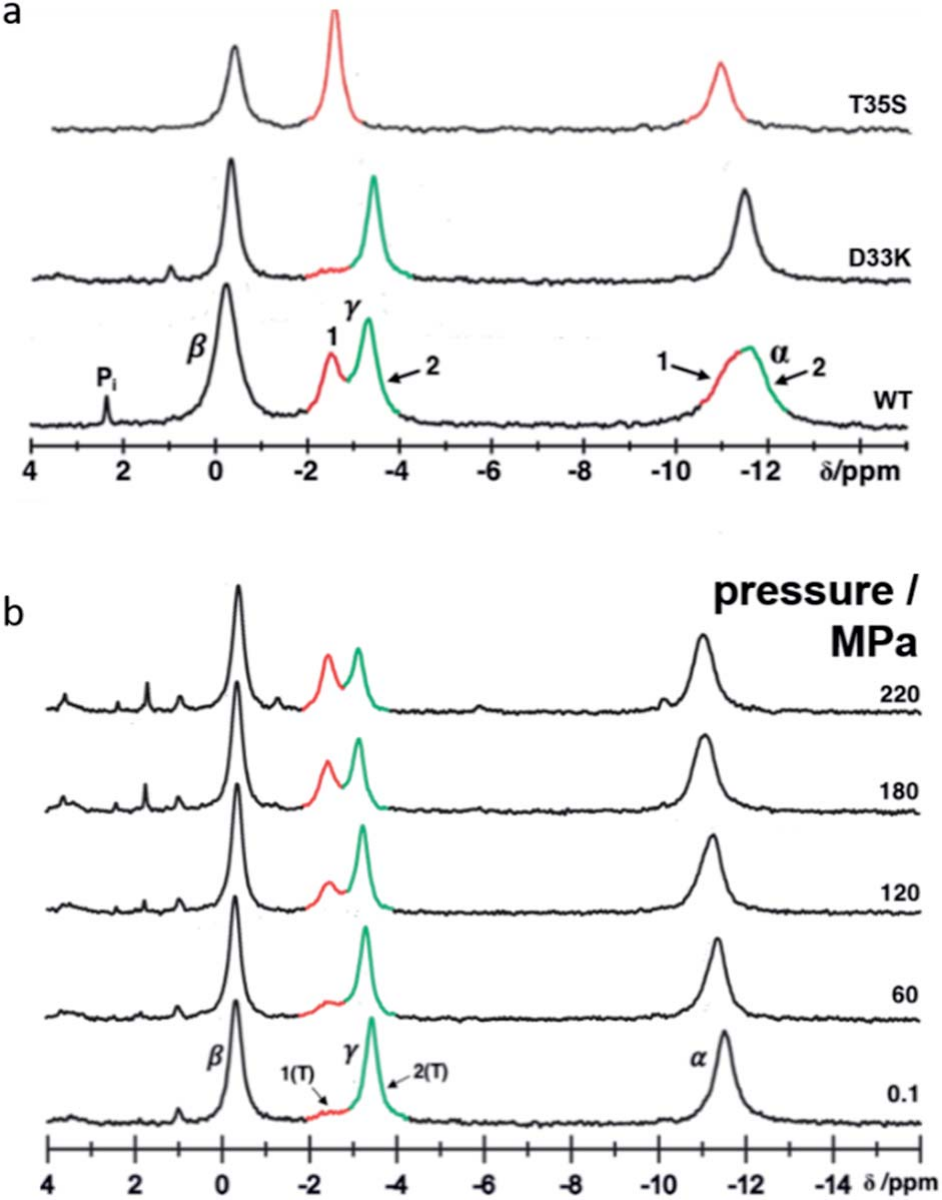
Conformational equilibria detected in Ras(D33K) by ^31^P NMR. (a) ^31^P NMR spectra of Ras(wt), Ras(D33K) and the state 1(T) mutant Ras(T35S) at 278 K and ambient pressure. Resonances corresponding to the α-, β- and γ-phosphate are indicated and the conformational states 1 and 2 are shown in red and green respectively (b) pressure-induced conformational shifts detected in Ras(D33K) by ^31^P HP-NMR at 278 K.

In solution the Asp 33 residue of ^15^N enriched Ras(wt) showed a pressure response that indicated that a transition is modified where state 2(T) is significantly involved.^12^ Therefore, the negatively charged Asp was replaced by the positively charged residue Lys. Relative to the wild-type protein the energy difference between state 1(T) and state 2(T) is strongly increased, in the ^31^P NMR spectra of Ras(D33K). The resonance corresponding to state 1(T) is almost invisible at ambient pressure (Fig. 1). Under pressure the state 2(T) is strongly stabilized relatively to Ras(wt) in which at higher pressures the state 1(T) dominates. The free energy difference Δ*G* between state 1(T) and 2(T) at ambient pressure and 278 K obtained from the HP-NMR experiments are 1.5 kJ mol^−1^ and 3.4 kJ mol^−1^ for Ras(wt)^12^ and Ras(D33K), respectively. Thus Ras(D33K) is a mutant that is predominantly in the effector binding state 2(T), in contrast to the wild-type protein.

### Crystal structure of Ras(wt) and Ras(D33K) at ambient pressure

To minimize potential experimental bias and to provide accurate comparisons between structures, we have first solved a reference structure of Ras(wt) in complex with Mg^2+^·GppNHp at room temperature (RT) and ambient pressure (AP) in the same experimental set-up and environments. As to be expected, its overall structure (ESI Fig. S1†) is similar to the previously reported Ras(wt) structures,^38–40^ the closest one being logically the RT structure corresponding to the PDB file *3tgp*. The phosphate binding loop, the switch I region, the G4 and G5 motifs involved in nucleotide binding are well defined. In contrast, the switch II region is mobile with high thermal *B*-factors. The switch II has indeed always been described as very flexible, with many alternate conformations, consistent with the hypothesis that this flexibility would be the rate limiting step of the GTP hydrolysis.^37,38^ The Mg^2+^-ion is, as expected, hexa-coordinated with one oxygen each from the β- and γ-phosphates, the two side chain hydroxyl groups of Ser 17 (from the P-loop) and Thr 35 (from the switch I) and two structural water molecules, termed here W1 and W2. This confirms that it mainly corresponds to state 2(T), the dominant state at ambient pressure.^12^

The crystal structure of Ras(D33K) in complex with Mg^2+^.GppNHp is almost identical to our Ras(wt) structure with a r.m.s.d. of the Cα atoms of 0.13 Å and similar average *B*-factors, which is not surprising since the equilibrium between state 1(T) and state 2(T) in solution is abolished within the crystal. The substitution of Asp 33 by a lysine does not induce significant structural modifications except a new hydrogen-bond between Lys 33 and Glu 31.

### Unit cell compressibility curves analysis

Analysis of the unit cell compressibility curves for Ras(wt) and Ras(D33K) indicates that the unit cell volume decreases almost linearly with pressure up to 300 MPa. A continuous rise of pressure above 300 MPa leads invariably to a complete loss of diffraction. However, nice diffracting crystals were unexpectedly recovered for pressures above 300 MPa after an overnight incubation at approximately 200 MPa. Associated with an improvement in the quality of diffraction, the unit cell compressibility curve then shows an expansion of the cell volume by 0.6% and 0.75% at 500 and 650 MPa, respectively compared to the 270 MPa unit cell volume for Ras(wt), indicative of a pressure-driven transition between sub-states (ESI Fig. S2†).

### Crystal structures of Ras(wt) and Ras(D33K) below and above the pressure-driven transition

**Fig. 2:**
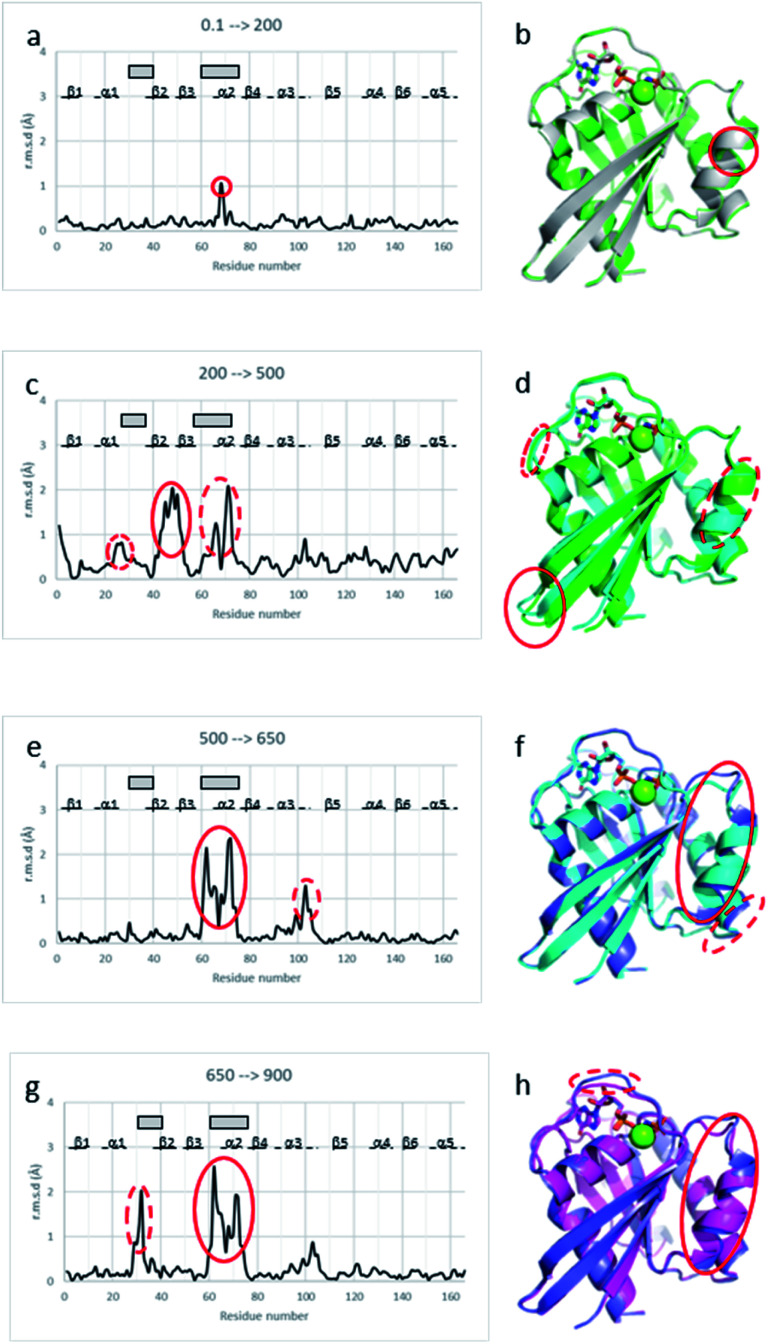
Structural differences between Ras structures at different pressure. Cα r.m.s. deviations between (a) Ras(wt) structures at 0.1 MPa and at 200 MPa, (c) Ras(wt) structures at 200 MPa and at 500 MPa, (e) Ras(wt) structures at 500 MPa and at 650 MPa, (g) Ras(wt) structure at 650 MPa and Ras(D33K) structure at 900 MPa. β-Strands are shown with lines, α-helices with dashed lines, and the switch I and II with grey rectangles. Structural superimpositions of (b) Ras(wt) at ambient pressure (grey) and 200 MPa pressure (green), (d) Ras(wt) at 200 MPa pressure (green) and 500 MPa pressure (cyan), (f) Ras(wt) at 500 MPa pressure (cyan) and 650 MPa pressure (slate), (h) Ras(wt) at 650 MPa pressure (slate) and Ras(D33K) at 900 MPa pressure (pink). Ras is shown in cartoon representation with GppNHp in stick representation coloured by atom types, the Mg^2+^-ion shown with a green sphere. The pressure-induced major shifts are shown both on graphs and respective structure representations with corresponding red ellipses.

Below the transition, the structural differences between the Ras(wt) or Ras(D33K) structures at 200 MPa and their corresponding structures at ambient pressure are small (r.m.s.d. 0.17 Å), the main differences occurring in the middle of helix α2 (Arg 68–Asp 69) that is part of switch II (Fig. 2a and b). Accordingly, there is no significant difference between Ras(wt) and Ras(D33K) at 200 MPa (r.m.s.d of 0.11 Å). In Ras(wt) at 200 MPa, *B*-factors strongly increase, especially in the switch II, while there is almost no increase in *B*-factors in Ras(D33K) (ESI Fig. S3†). A pressure of 200 MPa seems to induce a destabilization of Ras(wt) with a degradation in diffraction quality and a large increase of *B*-factors while Ras(D33K) seems to be less pressure-sensitive. The structure of Ras(wt) at 270 MPa is similar to the one at 200 MPa, but with a lower quality of diffraction, perhaps indicative of the beginning of a transition between sub-states at this pressure.

Above the transition, the improvement of the diffraction quality is confirmed by structure refinements as the number of visible water molecules increases, and the thermal *B*-factors significantly decrease in both Ras(wt) and Ras(D33K) (ESI Table S1 and Fig. S3†).

At 500 MPa, the average r.m.s.d on Cα atoms, relative to the 200 MPa structure becomes significantly larger than prior to the transition (r.m.s.d. of 0.49 Å), suggesting a clear structural rearrangement. Noticeably, there is a shift of the segment which precedes the switch I (Ile 24–Phe 28), a large shift of more than 1 Å of the switch II (Ser 65–Met 67, Gln 70–Met 72) and a larger shift of almost 2 Å of the β2–β3 loop (Ile 46–Leu 52) toward the helix α5 at the back of the molecule (Fig. 2c and d).

At 650 MPa, the zones that are mainly affected by pressure are similar to those at 500 MPa (r.m.s.d. between these two structures 0.28 Å), except in the switch II (Gln 61–Ala 66, Arg 68–Thr 74) which moves toward helix α3, shifting the end of this helix α3 (Arg 102–Asp 105) by around 1 Å (Fig. 2e and f).

In the structure of Ras(D33K) at 900 MPa, the zones that are mainly affected by pressure are similar to those at 650 MPa (r.m.s.d. between these two structures 0.33 Å), except in the beginning of switch I (Val 29–Asp 33), shifted by more than 1.5 Å toward the nucleotide and in the switch II (Gln 61–Ala 66, Arg 68–Arg73), shifting again the end of helix α3 (Fig. 2g and h).

The analysis of average thermal *B*-factors of Ras(wt) revealed that they all decreased in average by 12 Å^2^ at 500 MPa when compared to those at 200 MPa and decreased again in average by 6 Å^2^ at 650 MPa when compared to those at 500 MPa. Similarly, in Ras(D33K), the *B*-factors decreased by 10 Å^2^ at 900 MPa when compared to those at 200 MPa (ESI Fig. S3†).

The number of water molecules visible in the electron density map significantly increases between Ras(wt) at 500 and 650 MPa, and especially in Ras(D33K) between 200 and 900 MPa (ESI Table S1†). This reflects the hydration of the proteins by pressure,^3,6,8^ as similarly observed in many high pressure crystal structures,^46–48^ and may probably participate to the water-mediated allosteric interactions.^33^

In all structures of Ras(wt) and Ras(D33K) under pressure, there is almost no structural modification in the P-loop which tightly binds to the phosphate groups and contains the crucial Lys 16 and Ser 17 residues, as well as in the G4 and G5 motifs, both connected to the purine moiety of the nucleotide. The intrinsic rigidity of these regions, revealed by pressure, is therefore directly connected to the Ras cycling efficiency.

### Comparison of representative Ras structures in different structural states

In order to associate the structural determinants related to major Ras state transitions, we have compared representative structures of characterized Ras states to be able to thoroughly analyse the observed pressure-induced modifications.

The comparison of representative Ras structures in state 2(T) and in state 1(T) leads to a r.m.s.d value of 0.73 Å with two large displacements of more than 5 Å involving the switch I (Asp 30–Asp 38) which shifts away from the nucleotide, displacing the Thr 35 far from the Mg^2+^ ion, and the switch II (Gln 61–Arg 68, Gln 70–Arg 73) which shifts toward helix α3 (ESI Fig. S4a and b†).

The comparison of Ras structures in state 2(T) and in state 2(T)*-RBD leads to a r.m.s.d value of 0.43 Å and shows a shift by more than 1 Å toward the nucleotide of the switch I (Tyr 32–Pro 34), a large shift of around 2 Å of the first half of the switch II (Gln 61–Met 67), and a shift of around 1 Å of the β2–β3 loop (Arg 41–Cys 51) (ESI Fig. S4c and d†). These three segments are involved in binding to the regulatory site of the exchange factor SOS.

The structure of Ras in state 2(T)*-RBD differs from the structures of Ras in state 1(T) (r.m.s.d value of 0.73 Å) and in state 1(0) (r.m.s.d value of 2.3 Å), with large displacements in the switch I and in the switch II, and in a lesser extend in the C-terminal region, except in the loop β5–α4 for state 1(0) (ESI Fig. S4e–h†).

### Transition between states of Ras highlighted by pressure

**Fig. 3:**
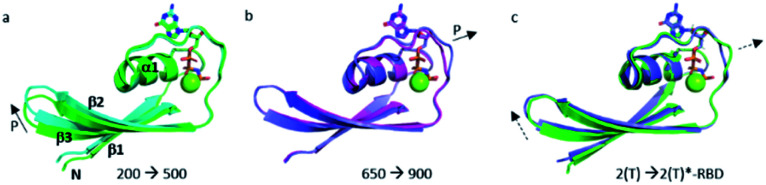
Structural differences in the N-terminal segment (Met 1–Thr 58). (a) Ras(wt) structures at 200 MPa (in green) and at 500 MPa (in cyan), (b) Ras(wt) structure at 650 MPa (in slate) and Ras(D33K) structure at 900 MPa (in pink), (c) PDB structures of Ras(wt) (*3tgp*, in green) and of Ras(Y64A) bound to the regulatory site of SOS (*1nvv* chain Q, in slate). Solid arrows show the direction of displacement when pressure increases, and dashed arrows show the direction of displacement from one state to another.

The structural shift of the N-terminal segment between Met 1 and Thr 58 encompassing β1, α1, β2 and β3, is similar in the transition from 200 MPa to 500 MPa, and in the transition between state 2(T) and 2(T)*-RBD, except in the loop Asp 30–Thr 35. This structure of the N-terminal segment does not further change at higher pressure, except at 900 MPa where the loop Asp 30–Thr 35 is shifted to the position observed in state 2(T)*-RBD (Fig. 3 and ESI Fig. S5a and b†).

The beginning of switch II (Ala 59–Arg 68) is always highly mobile with high *B*-factors in all structures considered here. At 200 MPa, the segment 59–68 is rather similar to its corresponding segment in state 2(T). It was previously shown that mutations in the AGQ motif (Ala 59–Gln 61) which modify its flexibility lead to conformations similar to transient intermediates in the Ras cycling process.^49,50^ At 500, 650 and 900 MPa, the segment 59–68 adopts different conformations which could correspond to transient intermediates termed here *I*_1_, *I*_2_ and *I*_3_, as observed in the mutants A59G and G60A.^49^

**Fig. 4:**
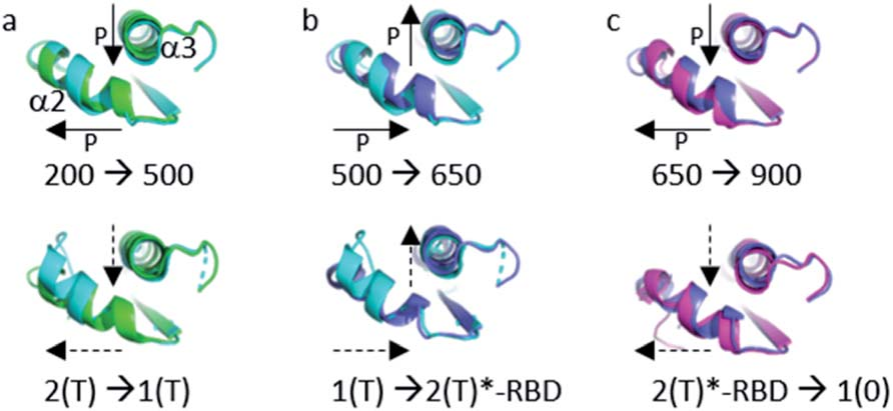
Structural differences in the segment Ala 59–Val 109. (a) (top) Ras(wt) structures at 200 MPa (green) and at 500 MPa (cyan) (bottom) *3tgp* (green) and *5b30* (cyan). (b) (top) Ras(wt) structures at 500 MPa (cyan) and at 650 MPa (slate) (bottom) *5b30* (cyan) and *1nvv* chain Q (slate). (c) (top) Ras(wt) structure at 650 MPa (slate) and Ras(D33K) structure at 900 MPa (pink) (bottom) Ras bound to the regulatory (slate) and catalytic (pink) sites in the ternary complex Ras(Y64A).GppNp:SOS:Ras(wt) (*1nvv* chain Q and chain R). Solid arrows show the direction of displacement when pressure increases, and dashed arrows show the direction of displacement from one state to another.

The structural shift in the segment from Asp 69 to Val 109, encompassing the end of α2, β4 and α3, is similar in the transition from 200 MPa to 500 MPa, and in the transition between state 2(T) and state 1(T). This segment is then similarly shifted in the transition from 500 MPa to 650 MPa, and in the transition between state 1(T) and state 2(T)*-RBD. At last, this segment is similarly shifted in the transition from 650 MPa to 900 MPa, and in the transition between state 2(T)*-RBD and state 1(0) (ESI Fig. S5c–e†). There is a correlated displacement of the second half of helix α2 and the end of helix α3 back and forth between two positions (Fig. 4).

There are only slight structural changes in the C-terminal domain from Pro 110 to His 166, encompassing β5, α4, β6 and α5, in the transition from 200 MPa to 500 MPa and in the transition between state 2(T) and state 2(T)*-RBD. There are almost no further changes in this segment at higher pressures (ESI Fig. S5f and S6†).

**Fig. 5:**
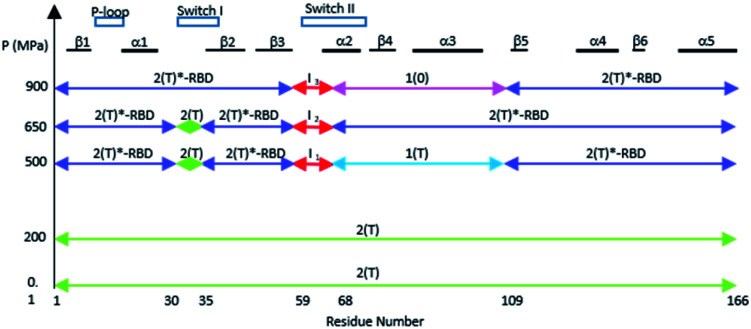
A schematic survey of the allosteric structural states within the three-dimensional structure of Ras at different pressures. The similarity with a state 2(T) structure is represented by a green arrow, with a state 2(T)*-RBD structure with a slate arrow, with a state 1(T) structure with a cyan arrow, and with a state 1(0) structure with a pink arrow. The intermediates states I1 to I3 in the beginning of switch II are represented with red arrows.

The different equilibria between structural states induced by pressure are schematized on Fig. 5.

### Allosteric network of Ras highlighted by pressure

Noticeably, the first (up to Arg 68) and second half (from Asp 69) of the switch II always behave differently whatever the transition between states or between structures at different pressure. Moreover, Arg 68 and Asp 69 are the only one modified between ambient pressure and 200 MPa (Fig. 2a and b). The switch II hinges around these two central residues with pressure. Such a hinge also occurs in the transition between states 2(T), 2(T)*-RBD, state 1(T) and state 1(0).

The allosteric interaction that has been described between the two lobes of Ras, the effector lobe (Met 1–Asn 86) and the allosteric lobe (Thr 87–His 166), has thus been evidenced in the different high-pressure structures, even if the limit between the two lobes are slightly different. The residues Asp 69–Asn 86 (in cyan on ESI Fig. S7a†) were assigned to the effector lobe while our study suggest that they would belong to the allosteric one.^34,36,37^

It was shown that the allosteric switch composed of helix α3 and the loop α3–β5 (residues Thr 87–Val 109) was shifted toward the helix α4 in the “on” state and toward the switch II in the “off” state (ESI Fig. S7b†).^26,36,37,51^ The “on” state was induced in the crystal by calcium acetate binding in the allosteric pocket between helices α3 and α4, while this pocket remains empty in the “off” state. As described above, the pressure-induced structural shift in the segment from Asp 69 to Val 109 moves back and forth between two positions of the end of helix α2 and the end of helix α3. The allosteric switch is shifted toward the helix α4 at 200 and 650 MPa as in the “on” state, while it is shifted toward the switch II at 500 and 900 MPa, as in the “ordered off” state since the switch II remains ordered (Fig. 4). The effect of pressure on Ras thus mimics a back-and-forth transition between the two allosteric states “on” and “ordered off”, while the allosteric pocket remains always empty, without any exogenous ligand.

It has been recently shown that Ras dimerization which occurs when Ras is bound to the membrane involves helices α4 and α5, belonging to the allosteric lobe.^52^ This area is not affected by pressure (ESI Fig. S5†), highlighting its intrinsic stability throughout the different conformational states.

## Discussion

In solution, the Gibbs free energy difference between the ground state 2(T) and the excited functional state with highest energy 1(0) is only 12.4 kJ mol^−1^ at atmospheric pressure. The same is true for the volume difference between these two states that are only −115 mL mol^−1^ corresponding to the size of approximately 6 water molecules. The free energy difference is even smaller between the ground state and the first exited state 1(T) with 1.5 kJ mol^−1^.^10,12^ These energies are modified significantly by pressure according to the partial molar volumes of the different states. Besides of these intrinsic free energy contributions of individual protein molecules, the energy contribution of the crystalline lattice influences the equilibrium, which is again pressure dependent. At ambient pressure, the equilibrium between structural state 2(T) and state 1(T) observed in solution is abolished in the crystal.^39^ Upon continuous pressurization above 300 MPa, a rapid conformational equilibrium between different conformational states as observed in solution leading to state 1(T) then to 2(T)*-RBD would cause a mixture of different conformations within the unit cell, leading to non-homogeneous crystals and to loss of long-range order resulting in poor diffracting crystals.

However, because of cooperative effects in the crystal lattice, a slow switch from a conformation to another (phase transition) is possible if at a given pressure a new stable conformer becomes dominant and compatible with the initial crystal packing. In such a situation, the diffraction quality will be restored and even improved after a long incubation time. Such a phase transition is likely to be observed in the pressure range between 300 and 400 MPa where diffraction was initially lost. The improvement in the quality of diffraction and the clear stabilization of the high pressure structures indicate that they may correspond to some relevant intrinsic conformational states of Ras.

The conserved water molecules W1 and W2 which coordinate the Mg^2+^ ion are always present whatever the pressure. The nucleotide, the Mg^2+^ ion, W1 and W2 have significantly lower *B*-factors than the average *B*-factors in all structures up to 650 MPa, revealing that the Mg^2+^ ion coordination is rigidified by pressure, suggesting its intrinsic stability. However, in the structure at 900 MPa, the *B*-factors of the nucleotide, the Mg^2+^ ion, W1 and W2 are higher than at ambient pressure, suggesting that this conformer may be indicative of a future release of the nucleotide, and a shift toward state 1(0) conformer.

Gln 61 is essential for GTP hydrolysis since its mutations to any other residue (except Glu) inhibits hydrolysis.^26^ In our structures of Ras(wt) and Ras(D33K) at ambient pressure, Gln 61 is oriented toward the nucleotide while this residue is usually oriented toward a symmetric molecule in the crystalline packing. Interestingly, a molecule of PEG is clearly visible in the electron density map of the Ras(wt) and Ras(D33K) structures whatever the pressure, between the switch II, the α3 helix and the α3* helix from a symmetric molecule (ESI Fig. S8†). Indeed, the crystals of Ras have to be stabilized in the diamond anvil cell (DAC) by increasing the concentration of PEG 400 to approximately 60%. It was previously shown that a high concentration of PEG or the binding of reducing agents like dithiothreitol in this pocket induce an ordered conformation of the switch II, in the “ordered off” state.^36^ Accordingly, the bound molecule of PEG seems to prevent in the 500 and 900 MPa structures the order-to-disorder transition of the switch II usually found in the “off” state.

Interestingly, in a structure of Ras in complex with the transition state analogue GDP–AIF_3_ (pdb file *1wq1*),^20^ which could be close to a GAP-interacting state 3(T) conformer,^12^ Gln 61 is also oriented toward the nucleotide. It has been proposed that this orientation of Gln 61 occurs in the transition state of the GAP-catalysed GTP hydrolysis.^40^

Gln 61 was proposed based on simulation studies to form strong hydrogen bonds with the inorganic phosphate in the complex Ras·GDP·Mg^2+^, stabilizing the hydrolysis intermediate.^53^ The orientation of Gln 61 in our structures up to 500 MPa could thus correspond to its position both in the complex with GAP and in the hydrolysis intermediate substates.

Our Ras(wt) structure at ambient pressure is probably closer to a state 2(T) structure than to a (still unknown) possible state 3(T) structure with the exception of the Gln 61 side chain, locally shifted as in a state 3(T) conformer. Interestingly, in the RT structure of Ras(wt) (pdb file *3tgp*), there are two alternate positions for Gln 61, a major one toward a symmetric molecule, and a minor one toward the nucleotide (ESI Fig. S9†).

The catalytic water molecule termed here W3 is stabilized by Gln 61 during GTP hydrolysis.^38–40^ This water molecule is visible in the structures at ambient, 500 and 650 MPa but not at 200 MPa, likely due to the overall increase of *B*-factors at this pressure. At 650 MPa, Gln 61 is oriented exactly as in state 2(T) structures stabilizing the catalytic water molecule W3, even if a PEG molecule is still bound in the interfacial pocket. Interestingly, in the most hydrated structure at 900 MPa, W3 disappears. In this structure Gln 61 switches to an intermediate position found in the constitutively active mutant Q61L (pdb file *3oiu*) or in the structure of Ras in complex with an effector (pdb file *4g0n*) (ESI Fig. S9†). The disappearance of the catalytic water molecule would suggest that the 900 MPa structure locally mimics an intermediate state like in the mutant Q61L.^51^

**Fig. 6:**
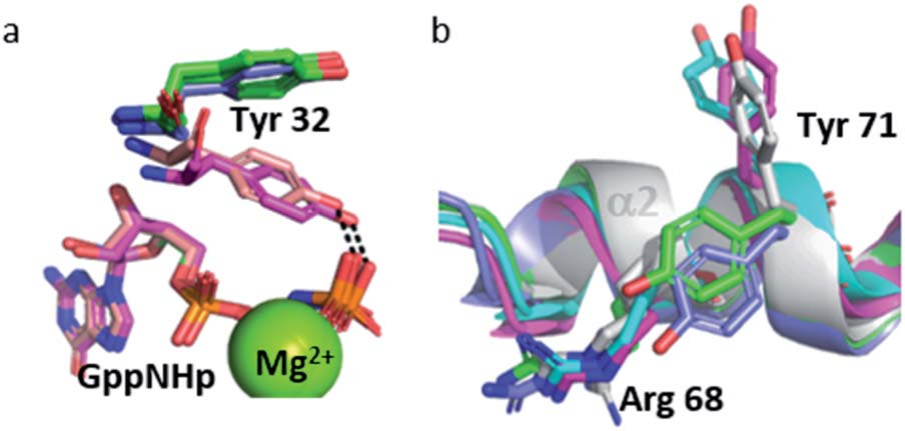
(a) Tyr 32 and GppNHp in stick representation in Ras(wt) at 650 MPa (slate) and 900 MPa (pink), in *3tgp* (green) and *3oiu* (salmon). (b) Cartoon representation of segment Gln 61–Thr 74 with residue Arg 68 and Tyr 71 in stick representation in Ras(wt) at 0.1 MPa (white), at 200 MPa (green), at 500 MPa (cyan), at 650 MPa (slate) and Ras(D33K) at 900 MPa (pink).

The Tyr 32 side chain belonging to the switch I either points toward the symmetrical γ-phosphate in state 2(T) Ras structures which crystallized in *P*3_2_21 space group or binds to a water molecule which bridges Tyr 32 to the γ-phosphate in state 2(T) Ras structures which crystallized in *R*32 space group.^38,39,54^ Tyr 32 is also bound to the nucleotide through a water molecule in the complexes of Ras with Ras-binding domains, like in Ras(Y64A)·Mg^2+^·GppNHp bound to the regulatory site of SOS that we call here 2(T)*-RBD.^37,45^ Up to the pressure of 650 MPa, Tyr 32 points toward the symmetrical γ-phosphate like in all state 2(T) Ras structures which crystallized in *P*3_2_21 space group. Interestingly, in Ras(D33K) at 900 MPa, Tyr 32 points toward the γ-phosphate of the nucleotide of the same subunit, in accord with our analysis which showed that at 900 MPa the segment Asp 30–Thr 35 is shifted toward its position in state 2(T)*-RBD. However, in Ras(D33K) at 900 MPa, Tyr 32 makes a direct H-bond to the γ-phosphate of the nucleotide, without any bridging water molecule. It has the same position than one of the two alternate positions of Tyr 32 observed in state 1(T).^42^ Interestingly, this orientation of Tyr 32 is also found in the structures of the constitutively active mutants G12V and Q61L (pdb files *3oiw* and *3oiu* respectively, Fig. 6a).^26,37,51^ It is worth noting that these two orientations of Tyr 32, either “in” or “out”, was also proposed based on FTIR experiments and simulations studies.^55^

Interestingly, the Tyr 71 residue seems to play a sensor role during the transition between the different states. This residue belongs to the second half of helix α2 whose main chain is displaced by pressure back and forth between two positions. At ambient pressure, its side chain is rotated toward the bulk. At 200 MPa, Tyr 71 side chain toggles by almost 180° to lie parallel to Arg 68 side chain, at a similar position than in state 2(T) structures. At 500 MPa, it toggles back toward the exterior, ready to interact with GEF, as it is the case in state 1(T) structures. At 650 MPa, it toggles again by almost 180° to lie parallel to Arg 68 side chain, in the same position than when Ras interacts with SOS in the state 2(T)*-RBD. And finally, at 900 MPa, it is again oriented toward the bulk, as it is the case in the nucleotide-free Ras structure (Fig. 6b). The side chain orientations of Tyr 71 are in accord with our analysis of conformer transitions within the segment Asp 69–Val 109, from state 2(T) at 200 MPa to state 1(T) at 500 MPa, then state 2(T)*-RBD at 650 MPa followed by state 1(0) at 900 MPa (Fig. 5). The role of sensor of Tyr 71 was previously suggested using the two mutants Q61G and A59G mutants, switching from a buried position which reinforces the interaction with the switch I to a solvent-exposed position in the transient GTP intermediates.^50^ Our different high-pressure structures confirm this back-and-forth motion between a buried and exposed position of the Tyr 71 residue.

The allosteric switch between the two lobes can be induced just using pressure instead of the binding of an exogenous molecule in the allosteric pocket, highlighting that pressure could mimic the allosteric effect of membrane binding to the allosteric lobe of Ras.^24,28^ Interestingly, increasing pressure can induce transition in both directions, oscillating between “on” and “off” allosteric states.

## Conclusion

Crystallography associated with high pressure perturbation (HPMX) is thus a very powerful tool to give insight into structural and dynamic behaviour of Ras, to visualize step by step within the 3D structure the modification of equilibrium between conformers, to determine the structural characteristics of high energy conformational states at atomic resolution which are very transient in solution, and to reveal the location in the protein sequence of excited states that might play important roles in the concerted motions between partners and would drive the transition between high-energy substates. It is thus complementary to HP-NMR which provides continuous pressure-induced shifts of the relative occurrence of the high-energy structures.

The very complex interplay between the switch I and switch II regions and the allosteric lobe are directly linked to the effector interaction and Ras cycling. *In crystallo* phase transition can be induced by pressure, opening the possibility to finely explore the structural determinants related to switch between Ras allosteric sub-states from a single sample type (*i.e.* without any mutations or exogenous partners). The multiplicity of sub-states, with many transient intermediates, the numerous water-mediated allosteric interactions between the two lobes, the multiple interactions between regulators and effectors, all these different substates and their interplay are mandatory for Ras function and can be deciphered by comparing the different Ras structures under pressure.

Ras explores many conformational substates depending of the different partners like regulators and effectors in relation with signalling pathways. Some of these conformers are weakly populated and difficult to experimentally characterize in ambient conditions. Pressure can thus have trapped these low populated conformers. The structures of the pressure-promoted conformers have revealed at the molecular level the sensor role of Tyr 71.

Finally, the present study paves the way to precisely describe Ras sub-states, and more generally to the description of G proteins sub-states, allowing the design of inhibitors specifically targeting the low populated conformers of Ras that have been promoted by pressure, and are likely to be involved during its interactions with partners.

## Experimental methods

### Protein expression and purification

The mutant Ras(D33K) was obtained from ptac vector presenting the wild type Ras protein (amino acids 1–166) by PCR based site directed mutagenesis using the Geneart® Site-Directed Mutagenesis kit, from Invitrogen® (Carlsbad, USA) according to the manufacturer's protocol. The mutated plasmids were used after the reaction to transform the highly competent DH5α™–TTR–*E. coli* cells, also provided in the same kit. Upon bacterial growth the plasmids were isolated and tested for the mutation through DNA sequencing using the Sanger method. Truncated variants (amino acids 1–166) of Ras(wt) and Ras(D33K) were expressed in LB medium in the *E. coli* strains CK600K and BL21, respectively using the pTac vector under the control of the *lac* operon. Upon IPTG induction, Ras was overexpressed overnight at 37 °C.^56^ Protein purification was accomplished in two steps, the first step being an anion exchange chromatography, using a Q-Sepharose column. A NaCl-based step-gradient was used for the elution of bound proteins. Fractions containing Ras were identified by SDS–PAGE and subsequently subjected to a size exclusion chromatography, using a Superdex 26/600 200 PG column (GE Healthcare, München, Germany). Both proteins were obtained in the GDP-bound form with a final purity >95% as judged by SDS–PAGE. Nucleotide exchange from GDP to GppNHp was subsequently performed at 278 K in a Mg^2+^-free buffer with addition of 200 mM (NH_4_)_2_SO_4_ and using a column with immobilized alkaline phosphatase (MoBiTec, Göttingen, Germany) in the presence of a 4-fold excess of GppNHp. After the reaction, free nucleotides, salt and other reaction by-products were removed by size exclusion chromatography using a PD10 column (Amersham, Freiburg, Germany). The concentration of the active, GppNHp-bound protein was determined by using a C18 reversed phase HPLC system equipped with a UV detector that was previously calibrated for the measurement of the guanine nucleotide at *λ* = 254 nm. The Ras protein was aliquoted in fractions of 500 μL and stored at 193 K in 40 mM Tris/HCl pH 7.5, 10 mM MgCl_2_, 2 mM DTE with a final concentration ranging between 1 to 1.5 mM. All chemicals used were of analytical grade and purchased from (Merck, Darmstadt, Sigma-Aldrich, Deisenhofen or Roche, Mannheim).

### High pressure NMR spectroscopy

Spectra were recorded with a Bruker Avance-600 spectrometer equipped with a Prodigy cold probe (Bruker) operating at a ^31^P frequency of 242.1 MHz. Pressure dependant ^31^P 1D spectra were recorded with proton decoupling during data acquisition by a GARP sequence^57^ with a *B*_1_-field strength of 980 Hz. High pressure NMR spectra were recorded using a home built online-pressure system (for details, see ref. 58), and a ceramic sample cell (with an outer diameter of 5 mm and an inner diameter of 3 mm) from Daedalus Innovations LLC (Aston, PA, USA). ^31^P NMR spectra were indirectly referenced to DSS with a *Ξ*-value of 0.4048073561 reported by Maurer and Kalbitzer^59^ which corresponds to 85% external phosphoric acid contained in a spherical bulb.

### Crystallization

HPMX requires the use of crystals, typically 100 μm to 400 μm in edge length. For that purpose, Ras(wt) or Ras(D33K) crystals were produced according to the “batch mode” methodology, using an appropriate crystallization chamber containing 3-spot wells made of glass that were previously siliconized. To each of these spots, typically 15–30 μL of 80 mM Tris–HCl pH 7.5, 20 mM MgCl_2_, 4 mM DTE and 52–60% PEG-400 were placed at their centre. 30 μL of the protein solution were then carefully added. The resulting solutions were slowly mixed by gentle pipetting. The chambers were immediately sealed with transparent adhesive tape and placed in a vibration-free zone and in the dark at room temperature. Nucleation spots were visible under the microscope after *ca.* 6 h and full crystals in trigonal space group *P*3_2_21 were grown after 2–3 days.

### Crystallographic data collection under high hydrostatic pressure

Ras(wt) or Ras(D33K) crystals were loaded into a diamond anvil cell (DAC), as previously described.^46,60,61^ The solution used as the compression medium consisted of the mother liquor with a higher concentration of PEG 400 (55 to 65%) to stabilize the crystals inside the DAC.

Diffraction data were recorded at room temperature on the ID09 and ID27 beamlines at the ESRF synchrotron (Grenoble, France) at wavelengths *λ* = 0.415 Å and *λ* = 0.374 Å respectively. Detectors were a MarResearch Mar555 flatpanel on ID09 and a MarResearch MarCCD165 on ID27. The pressure within the DAC compression chamber was monitored through the pressure-dependent fluorescence of a ruby chip used as an internal probe. Exposure times were 5 s and 30 s per frame respectively on ID09 and ID27 with an oscillation angle of 1°. Only one crystal was used for each data set, thanks to the large aperture DAC, specifically designed for HPMX.^61^ During data collections, the crystal was translated in the beam every 10° of rotation to limit the crystal degradation by irradiating fresh portions of the crystal.

### Structure determination, refinements, and analysis

All data sets were indexed and integrated using XDS.^62^ The integrated intensities were scaled and merged using SCALA and TRUNCATE and the structures were solved by molecular replacement (MR) with MOLREP, from the CCP4 package.^63^ In order to minimize any bias in the areas involved in the transition between states, MR's were performed using a model from the PDB file *5p21* (ref. 38) where the switch I (Asp 30–Arg 41) and switch II (Gly 60–Glu 76) were removed in addition to all heteroatoms and alternate positions. Refinements were carried out using REFMAC^64^ starting from the MR structure. All these programs are part of the CCP4 package.^63^ The graphic program Coot^65^ was used to visualize |2*F*_obs_ − *F*_calc_| and |*F*_obs_ − *F*_calc_| electron density maps and for manual rebuilding of switches I and II during the refinement steps. Water molecules were carefully introduced in the data only if they were at correct H-bond distances and with individual unrestrained thermal factor not exceeding 60 Å^2^.

The Cα r.m.s. deviations and the average backbone thermal *B*-factors were computed using programs from CCP4 package. A summary of data collection and refinements statistics is reported in ESI Table S1†. The figures have been made using PyMOL (version 2.4.0 Schrödinger, LLC).

## Data availability

The atomic coordinates and structure factors reported in this article have been deposited in the Protein Data Bank, with ID numbers *7og9*, *7oga*, *7ogb*, *7ogc* for Ras(wt) at 0.1 MPa, 200 MPa, 500 MPa and 650 MPa respectively, with ID numbers *7ogd*, *7oge*, *7ogf* for Ras(D33K) at 0.1 MPa, 200 MPa, and 900 MPa respectively.

## Author contributions

E. G., H. R. K and N. C. conceived the project, E. G., P. L., M. S., A. C. D., T. P., H. R. K. and N. C. contributed to the HPMX experiment, E. G., T. P., H. R. K. and N. C. analysed the data and wrote the manuscript.

## Conflicts of interest

There are no conflicts to declare.

## Supplementary Material

SC-013-D1SC05488K-s001
