# Supplementary material for: Equilibria between conformational states of the Ras oncogene protein revealed by high pressure crystallography
Source: Chem Sci. 2022 Jan 13;13(7):2001–10. doi: 10.1039/d1sc05488k (PMC8848853; doi:10.1039/d1sc05488k)
Supplement: SC-013-D1SC05488K-s001 [file SC-013-D1SC05488K-s001.pdf]

Electronic Supplementary Material (ESI) for Chemical Science.
This journal is © The Royal Society of Chemistry 2022

†Electronic Supporting Information

Equilibria between conformational states of the Ras oncogene protein revealed by high pressure crystallography

Eric Girard,[‡a] Pedro Lopes,[‡b] Michael Spoerner,[b] Anne-Claire Dhaussy,[c] Thierry Prangé,[d] Hans Robert Kalbitzer,*[b] and Nathalie Colloc'h*[e]

[a] Univ. Grenoble Alpes, CEA, CNRS, IBS, Grenoble, France
[b] Institute of Biophysics and Physical Biochemistry and Centre of Magnetic Resonance in Chemistry and Biomedicine, University of Regensburg, Regensburg, Germany, hans-robert.kalbitzer@biologie.uni-regensburg.de
[c] Normandie Univ,. Ensicaen, CNRS, CRISMAT UMR 6508, Caen, France
[d]CitCOM UMR 8038, CNRS, Université de Paris, Faculté de Pharmacie, Paris, France
[e]ISTCT UMR 6030, CNRS, Université de Caen Normandie, CERVOxy group, Centre Cyceron, Caen, France, colloch@cyceron.fr
‡These authors contributed equally

Content:

Table S1
Figures S1-S9

Table S1. Summary of data collection and refinement statistics

**Ras**	wt	wt	wt	wt	D33K	D33K	D33K
**PDB**	**7OG9**	**7OGA**	**7OGB**	**7OGC**	**7OGD**	**7OGE**	**7OGF**
**Data collection**							
Pressure (MPa)	0.1	200	490	650	0.1	200	880
Resolution (Å)	1.75	1.90	1.85	1.70	1.95	2.10	1.80
Cell dimensions							
a (Å)	39.75	39.48	38.63	38.9	39.79	39.57	38.42
c (Å)	161.80	160.81	166.14	165.09	161.70	160.65	165.4
Cell volume (nm^3)	221.403	217.084	214.711	216.347	221.712	217.842	211.437
Completeness (%)	99.4 (99.9)	98.7 (98.9)	99.3 (100)	99.0 (99.0)	87.9 (88.7)	95.2 (96)	92.5 (93.2)
Redundancy	4.8 (4.9)	4.6 (4.7)	4.9 (5.1)	4.5 (4.7)	5.8 (5.8)	5.6 (5.7)	5.7 (5.7)
Unique reflections	15666 (2243)	11950 (1678)	13092 (1848)	16670 (2353)	9920 (1426)	8578 (1235)	12669 (1816)
R_{merge} (%)	7.2 (70.1)	7.3 (74.8)	20.6 (77.4)	16.3 (102.6)	14.4 (84.6)	14.9 (70.5)	11.4 (52.1)
R_{pim} (%)	3.7 (34.5)	3.8 (37.9)	9.5 (34.5)	7.8 (47.7)	6.7 (37.9)	6.6 (31.1)	4.9 (23.3)
I/σ(I)	13.9 (1.8)	10.5 (1.8)	4.4 (1.8)	9.2 (2.2)	8.8 (1.7)	8.4 (2.4)	10.4 (2.5)
**Refinement**							
R_{work} (%)	15.83	17.11	17.77	16.05	18.07	17.25	24.19
R_{free} (%)	21.69	23.53	23.58	22.07	24.95	26.25	33.30
Mean standard deviation from ideality							
Length (Å)	0.0197	0.0179	0. 0195	0.0194	0.0162	0.0133	0.0157
Angle (°)	2.048	1.970	1.970	1.997	1.842	1.775	2.060
No water	103	81	106	163	82	81	210
No PEG molecule	1	1	3	3	1	1	3
Mean B factors (Å^2)							
Protein	27	37	23	18	27	29	18
Nucleotide	18	26	17	10	18	19	20
Mg^{2+}	17	25	17	9	16	21	21
Water	42	46	34	32	35	36	28
PEG	63	66	44	47	53	49	32

[a]Values in parentheses are for the highest resolution shell.

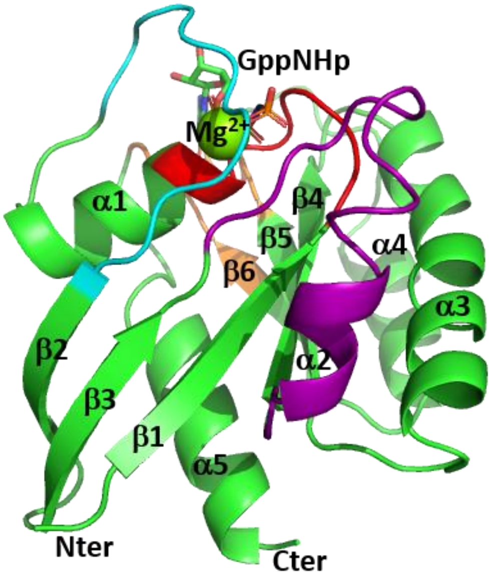

Figure S1. Overall structure of Ras.Mg^{2+}.GppNHp. Ras is shown in green in cartoon representation, with the P-loop (Gly 10 – Ser 17) in red, the switch I (Val 29 – Tyr 40) in cyan, the switch II (Asp 57 – Glu 76) in purple, the G4 (Asn 116 – Asp 119) and G5 (Glu 143 – Lys 147) motifs in orange, the GppNHp shown in stick representation coloured by atom types, and the Mg^{2+} ion shown with a green sphere.

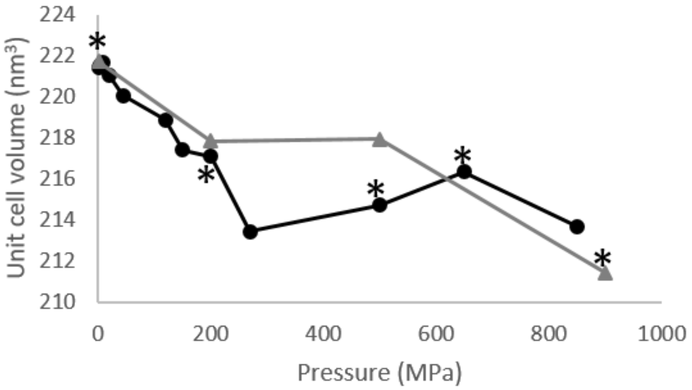

Figure S2. Unit cell compressibility curves. The metric dependencies of the crystal cell volumes *vs* the applied pressure are shown in black for Ras(wt) and in grey for Ras(D33K). The complete data sets that have been refined and discussed in the manuscript are shown with a star. The data set of Ras(wt) at 270 MPa was of lower diffraction quality than the one at 200 MPa (lower resolution and worse statistics). The data set of Ras(wt) at 850 MPa was at lower resolution (3.5 Å) and the data set of Ras(D33K) at 500 MPa was incomplete.

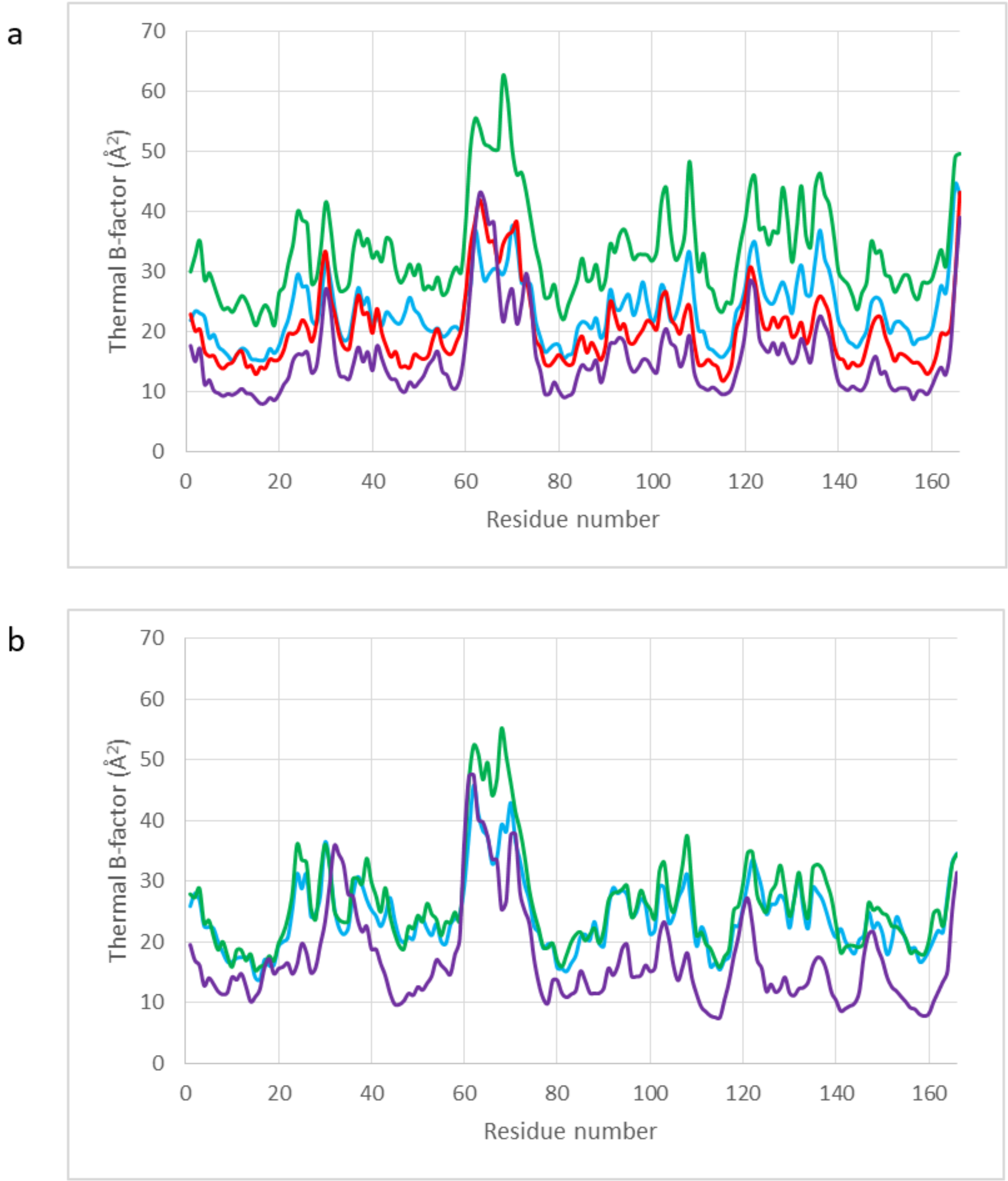

Figure S3. Backbone thermal B-factors analysis. (**a**) Ras(wt) structures at 0.1 MPa (in cyan), at 200 MPa (in green), at 500 MPa (in red) and at 650 MPa (in purple). (**b**) Ras(D33K) structures at 0.1 MPa (in cyan), at 200 MPa (in green), and at 900 MPa (in purple).

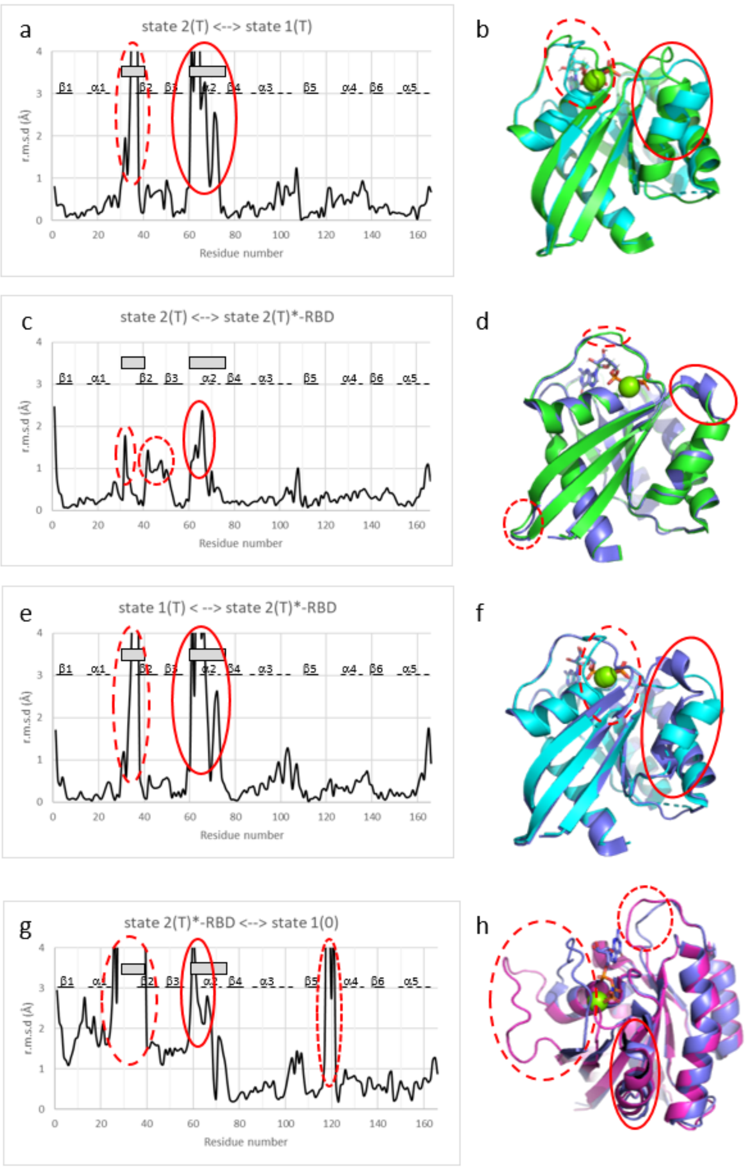

Figure S4. Structural differences between representative Ras structures in different states. $C\alpha$ r.m.s. deviations between (**a**) *3tgp* and *5b30,* representative structures of Ras in state 2(T) and in state 1(T) respectively, (**c**) *3tgp* and chain Q of *1nvv* (Ras(Y64A) of the Ras(Y64A).GppNp:SOS:Ras(wt) complex), representative structures of Ras in state 2(T) and in state 2(T)*-RBD respectively, (**e**) *5b30* and chain Q of *1nvv*, representative structures of Ras in state 1(T) and in state 2(T)*-RBD respectively, (**g**) chain Q and R of *1nvv*, representative structures of Ras in state 2(T)*-RBD and in state 1(0) respectively. β-strand are shown with lines, α-helix with dashed lines, and the switch I and II with grey rectangles. Structural superimposition of (**b**) *3tgp* (green) and *5b30* (cyan), (**d**) *3tgp* (green) and *1nvv* chain Q (slate), (**f**) *5b30* (cyan) and *1nvv* chain Q (slate), (**h**) *1nvv* chain Q (slate) and chain R (pink) (Ras bound to the regulatory and catalytic sites in the Ras(Y64A).GppNp:SOS:Ras(wt) complex respectively). Ras is shown in cartoon representation with GppNHp in stick representation coloured by atom types, the Mg^{2+}-ion shown with a green sphere. Significant pressure-induced changes are illustrated both on graphs and respective structure representations with corresponding red ellipses.

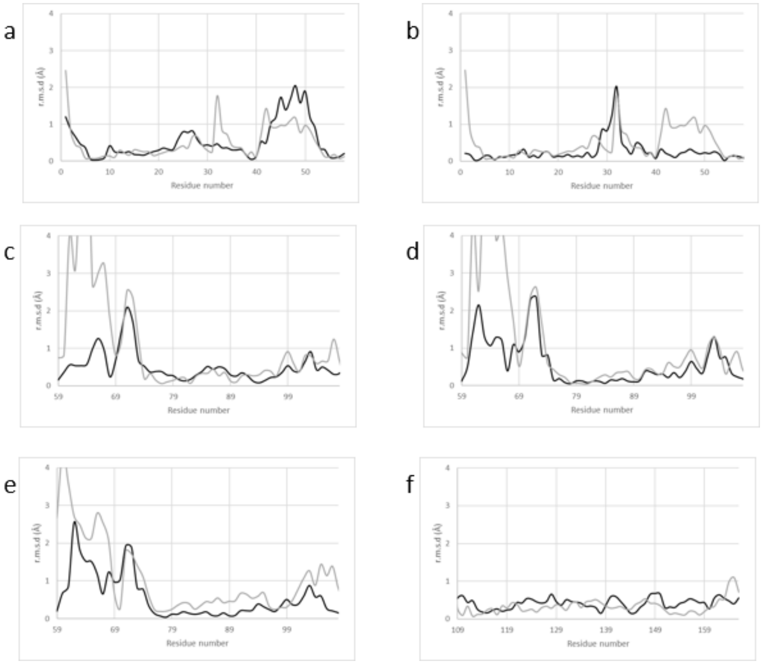

Figure S5. Cα r.m.s. deviations in the N-terminal segment (1-58) (**a**) between Ras(wt) structures at 200 MPa and at 500 MPa (black) and between *3gtp and* chain Q of *1nvv* (grey), (**b**) between Ras(wt) structures at 650 MPa and at 900 MPa (black) and between *3gtp and* chain Q of *1nvv* (grey). Cα r.m.s. deviations in the central segment (59 – 109) (**c**) between Ras(wt) structures at 200 MPa and at 500 MPa (black) and between *3gtp and 5b30* (grey), (**d**) between Ras(wt) structures at 500 MPa and at 650 MPa (black) and between *5b30* and chain Q of *1nvv* (grey), (**e**) between Ras(wt) structure at 650 MPa and Ras(D33K) structure at 900 MPa (black) and between chain Q and R of *1nvv* (grey). (**f**) Cα r.m.s. deviations in the C-terminal segment (110 – 166) between Ras(wt) structures at 200 MPa and at 500 MPa (black) and between *3gtp* and chain Q of *1nvv* (grey).

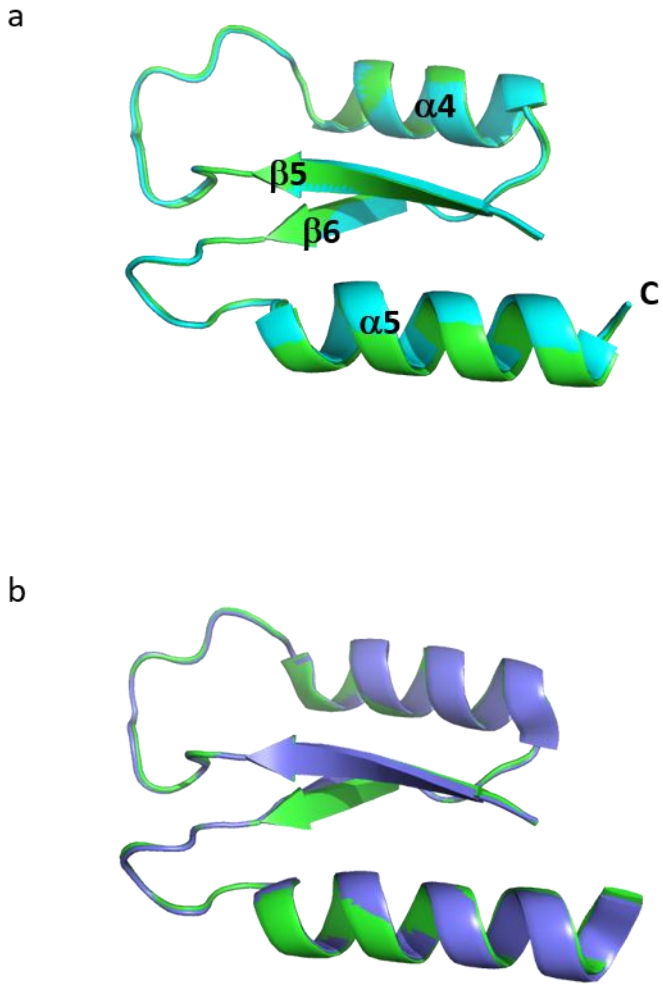

Figure S6. Structural differences in the C-terminal segment (Pro 110 – His 166). (**a)** Ras(wt) structures at 200 MPa (green) and at 500 MPa (cyan), (**b**) *3tgp* (green) and *1nvv* chain Q (slate).

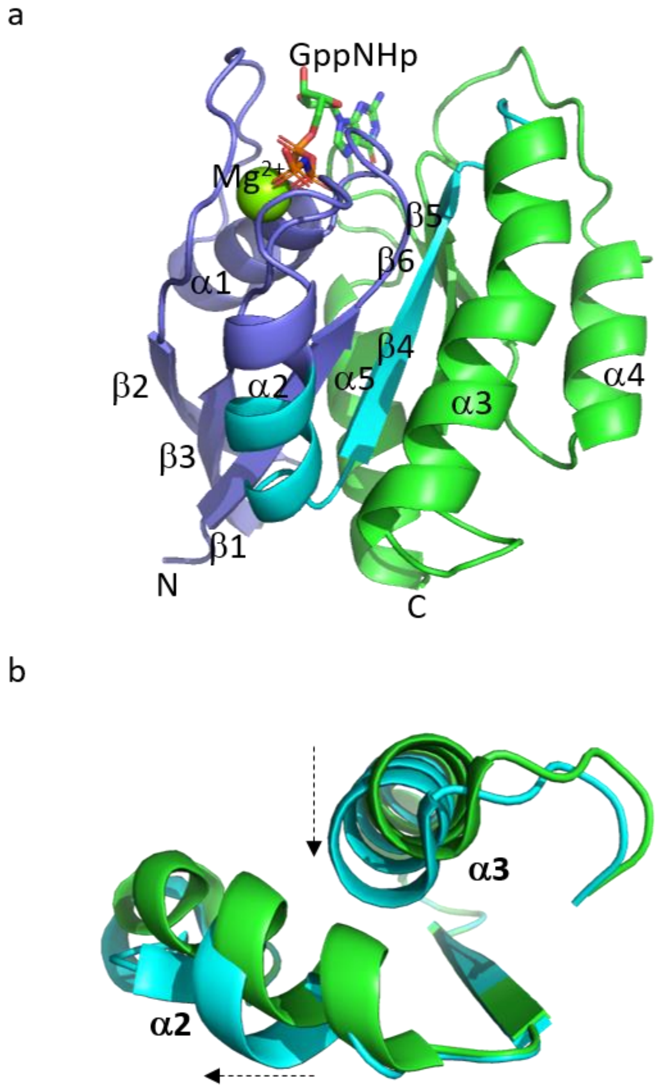

Figure S7. (a) Overall structure of Ras shown in cartoon representation, with the effector lobe coloured in slate (Met 1 – Arg 68) and cyan (Asp 69 – Asn 86) and the allosteric lobe (Thr 87 – His 166) in green, the GppNHP shown in stick representation coloured by atom types, and the Mg^{2+} ion shown with a green sphere. (**b)** Structural differences in the segment Ala 59 – Val 109 in a structure in the "on" state in green (PDB file *3k8y*) and in the "off" state in cyan (PDB file *2rge*). Dashed arrows show the direction of displacement from the "on" state to the "off" state.

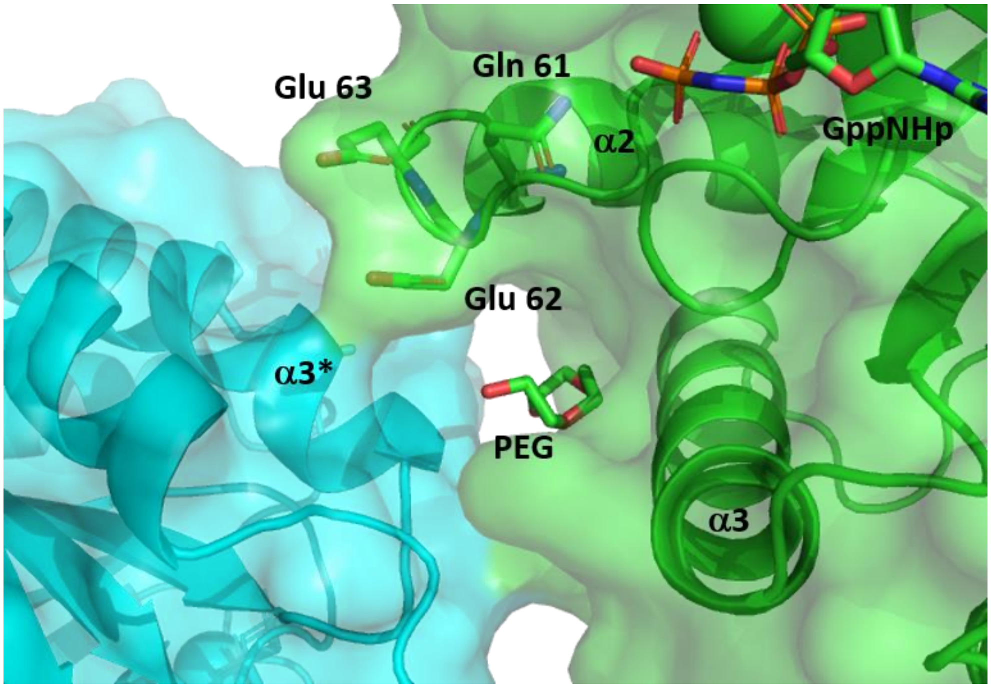

Figure S8. The PEG molecule binding site, located between the helix α3 (in green) and the helix α3* from a symmetric molecule (in cyan), at a distance of 4.5 Å of Glu 62 side chain, which in turn shifts the Glu 63 side chain orientation. This PEG molecule binds in all our ambient and high pressure structures of Ras(wt) and Ras(D33K).

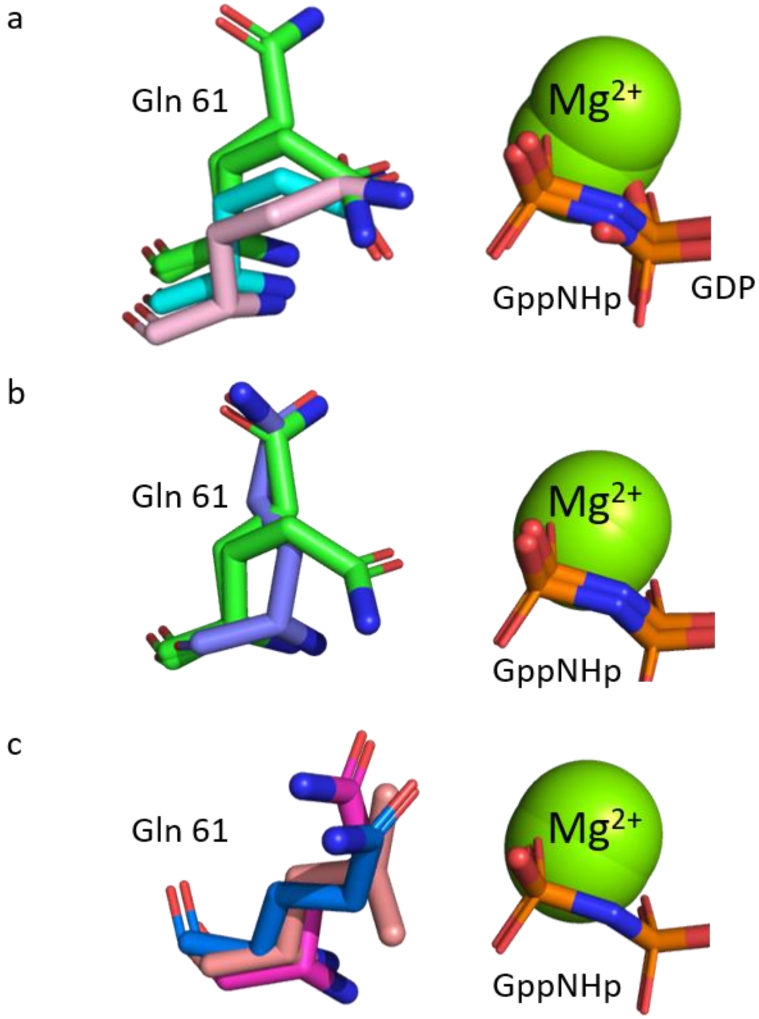

Figure S9. The different positions of Gln 61. **(a)** Gln 61 in Ras(wt) at 500 MPa (cyan), in *3tgp* (green) and in *1wq1* (light pink). **(b)** Gln 61 in Ras(wt) at 650 MPa (slate) and in *3tgp* (green). **(c)** Gln 61 in ras(wt) at 900 MPa (pink), in *3oiu* (salmon) and in *4g0n* (blue). The Gln 61 and GppNHp (or GDP in *1wq1*) are shown in stick representation with the Mg^{2+} with a green sphere.
